# Motivational Self-Regulation Strategies and Their Relationship with Academic Adjustment Across Adolescent High-Ability, Learning-Disability, and Regular Students

**DOI:** 10.3390/jintelligence14090202

**Published:** 2026-09-01

**Authors:** Zeltia Martínez-López, Mª Emma Mayo, Jose Eulogio Real, Carolina Tinajero

**Affiliations:** 1 Institute of Psychology (IPsiUS), University of Santiago de Compostela, 15782 Santiago de Compostela, Spain; emma.mayo@usc.es (M.E.M.); carolina.tinajero@usc.es (C.T.); 2 Department of Social Psychology and Methodology, University of Santiago de Compostela, 15782 Santiago de Compostela, Spain; joseeulogio.real@usc.es

**Keywords:** motivational self-regulation, academic adjustment, high abilities, learning disabilities

## Abstract

Motivational self-regulation strategies (MRSs) play a key role in how students initiate, sustain, and direct effort during learning. However, little is known about their use among adolescents with different educational profiles. This study aimed to explore possible differences in the use of MRSs among adolescent students with varying learning potential (high-ability, learning-disability and regular students) and to examine whether the relationship between MRSs and academic adjustment differed across these educational profiles. We recruited a sample of 2191 students enrolled in compulsory secondary education, comprising regular students (81.8%, *N* = 1792), students with learning disabilities (15.8%, *N* = 347), and high-ability students (2.4%, *N* = 52). The participants completed self-report measures of MRSs and academic adjustment. Path models were used to examine cross-sectional direct and indirect associations among the study variables, and they revealed different patterns across the three groups. High-ability status was directly and positively associated with academic adjustment, while learning-difficulty status showed a direct negative relationship with adjustment. The three groups of students showed indirect associations with adjustment via various types of MRSs.

## 1. Introduction

In recent decades, educational systems worldwide have become increasingly inclusive, reflecting a growing commitment to equity, diversity, and equal opportunities (UNESCO, 2024). This aspiration is reflected in Sustainable Development Goal 4 (SDG 4) of the United Nations 2030 Agenda for Sustainable Development (United Nations, 2015). It emphasizes meeting the needs of students with different learning potential, particularly those with specific educational needs (UNESCO, 2020). In this regard, promoting self-regulated learning has been proposed as a valuable approach, as it can improve the participation and success of students with different profiles by capitalizing on their individual strengths and addressing their specific learning needs (Efklides, 2019; Krämer et al., 2021). Intervention programs, whether they are aimed at students with learning disabilities or high-ability students who are underachieving, typically include training on self-regulated learning skills (Peña-San José et al., 2026; Perry et al., 2020), since both types of students tend to underutilize these skills compared to their peers (Kampylafka et al., 2023; Stoeger & Sontag, 2012). A further rationale for the implementation of these programs lies in the growing importance of self-regulated learning in contemporary society (OECD, 2018). To optimize the results of these programs, it is necessary to identify the differences among students with varying learning potentials in terms of their knowledge and their application of different types of learning strategies. The area of motivational self-regulation requires special attention, as it is significantly underexplored (Al-Hoorie, 2024; Efklides, 2019).

## 2. Self-Regulated Learning and Motivational Self-Regulation

Self-regulated learning is a process in which individuals self-generate their thoughts, feelings, and actions in order to deliberately and systematically achieve their goals (Pintrich, 2000; Zimmerman, 2000). Self-regulation comprises four different areas: (meta)cognition, behavior, context, and motivation (Pintrich, 2002). The motivation-related component has attracted increasing scholarly attention (Fong et al., 2023; Tinajero et al., 2024; Trautner et al., 2025), since motivation has been deemed essential for academic persistence and success (Miele & Scholer, 2016; Villar et al., 2024). Studies conducted to date have generally identified a positive relationship between motivational self-regulation and several indicators of academic progress, such as engagement and performance (Martínez-López et al., 2024; Grunschel et al., 2016; Smit et al., 2017; Suárez-Riveiro et al., 2016).

Motivational self-regulation refers to the process of assessing one’s motivational state and employing strategies to initiate, sustain, and/or enhance motivation (Grunschel et al., 2016; Wolters, 2003). Several theoretical models focused on motivational regulation have been proposed. Notably, in their Metamotivational Model of Motivation Regulation, Miele and Scholer (2016, 2018) identified task value (interest, importance, or potential utility) and self-efficacy as key dimensions of motivation that individuals commonly monitor and regulate. According to this model, initial motivation is driven by a higher-order goal (e.g., successfully completing a course) which subsequently directs the selection and pursuit of more specific task-related goals (e.g., passing an exam). The model also includes exogenous obstacles and costs (e.g., alternative actions that are waived) that can negatively affect motivational resources. Metamotivational feelings, such as frustration or enjoyment, emerge from individuals’ evaluations of these components, act as signals of their current motivational state, and may ultimately prompt learners with low motivation to withdraw from the activity or pursue a different objective. Conversely, learners can draw on their metamotivational feelings and knowledge, including their personal beliefs and theories about motivation, to determine the origins of the incentivization problem and select suitable motivational regulation strategies (MRSs) to strengthen task motivation or alleviate its associated costs.

### 2.1. Motivational Regulation Strategies

MRSs can be classified based on the motivational dimension they primarily target. Special emphasis has been placed on the self-management of academic goals, linked to both self-relevant and utility task value, as it is regarded as the foundation of self-regulated learning in any learning context (Miele & Scholer, 2018; Wolters, 2003). Students regulate their academic goals through a variety of goal-directed self-talk strategies (Wolters & Benzon, 2013). Specifically, *mastery self-talk* involves encouraging oneself to prioritize learning and skill development. To regulate performance goals, learners may engage in *personally referred self-talk*, which emphasizes personal improvement; *self-enhancing self-talk*, which focuses on demonstrating competence relative to others; or *self-defeating self-talk*, which aims to avoid negative evaluations from others. Additionally, *work-avoidance self-talk* is characterized by an emphasis on minimizing effort (Villar et al., 2024).

When self-regulating their motivation, students can also engage in *self-efficacy enhancement*, which involves reinforcing beliefs about one’s abilities and strengths; *enhancement of situational interest*, whereby learners attempt to increase the intrinsic appeal of a task by making it more engaging or enjoyable; *cost appraisal*, which consists of evaluating whether the effort and resources invested are justified by the anticipated academic benefits; and *self-consequating*, a strategy through which students reward themselves for task engagement, thereby attributing greater attainment value to academic activities (Villar et al., 2024).

### 2.2. Motivational Self-Regulation in High-Ability and Learning-Disability Students

Although it is accepted that the selection and application of MRSs depend on the student’s interpretation of their suitability for the specific task and learning situation at hand, some intraindividual consistency is also assumed (Miele & Scholer, 2018; Schwinger & Stiensmeier-Pelster, 2012). Research has examined whether variations in self-regulated learning moderate the motivation and academic outcomes of students with diverse learning potential. This research is driven by two main objectives: gaining a deeper understanding of the processes involved in the development and application of learning strategies and providing a basis for the design of effective and inclusive interventions aimed at fostering these skills (Aydan & Mamas, 2025; Baccassino & Pinnelli, 2023). Within this framework, some researchers have focused on contrasting learning-strategy use of regular students with that of high-ability students or those with learning disabilities (Aydan & Mamas, 2025; Kranjec & Bakračevič, 2023). The learning strategies that high-ability students in particular tend to use may contribute to the superior academic outcomes they commonly display, whereas the origin of the difficulties experienced by students with learning disabilities or underachieving high-ability students may lie in a deficient use of learning strategies.

High-ability students are those who perform at or exhibit the potential to perform at an outstanding level in one or more domains (Raoof et al., 2024). According to the Eurydice (2006), the prevalence of gifted children in European countries is estimated to be around 3–10% of the school population. Available evidence suggests that high-ability students exhibit higher levels of metacognitive awareness and possess more declarative knowledge about learning strategies than their peers. Similar trends have been observed in studies examining the use of learning strategies, although the findings are not entirely consistent and appear to depend on factors such as age, educational context, and the specific strategies under investigation (Stoeger & Sontag, 2012; Zeidner & Stoeger, 2019). Nevertheless, substantial gaps remain in the literature, particularly with respect to motivational self-regulation strategies, a topic that has received very limited empirical attention. To the best of our knowledge, the only published study on this topic is that of Zimmerman and Martinez-Pons (1990), in which a structured interview about self-regulated learning strategies was administered to gifted and regular 5th-, 8th-, and 11th-grade students. In that study, gifted participants reported a greater use of self-consequating than regular students.

Learning disabilities are understood as the various personal conditions that interfere with individuals’ ability to learn (Vida et al., 2024, p. 20). Students with these disabilities generally receive support within the education system through specific educational measures. In Spain, the country of origin of the sample used in this study, it is estimated that 19.8% of students in compulsory secondary education require such measures (Ministerio de Educación, Formación Profesional y Deportes, 2026). Empirical data show that students with learning disabilities report lower levels of self-regulation strategies than their non-disabled peers (Aydan & Mamas, 2025; Kampylafka et al., 2023). Although students with learning disabilities often face motivation problems (Wery & Thomson, 2013), their use of motivational learning strategies in comparison with non-disabled students remains underexplored. To the best of our knowledge, only one recent study by Gehle et al. (2023) has focused on this issue. The authors reported a broad use of self-consequating and self-efficacy self-talk among primary school students with special educational needs. Both strategies were positively associated with effort.

### 2.3. Motivational Self-Regulation and Academic Adjustment

Academic adjustment is conceived as the degree of fit attained by an individual regarding academic demands, as reflected in their attitude, engagement, and the adequacy of their study habits (Credé & Niehorster, 2012). Both gifted students and those with learning disabilities often feel that they do not fit into the educational system (Almukhambetova & Hernández-Torrano, 2020; Parpottas et al., 2023), which can affect their academic progress. Motivational self-regulation (considered globally) has been associated with different indicators of academic adjustment, such as adoption of (meta)cognitive learning strategies, positive academic emotions, effort, engagement, and achievement (Trautner & Schwinger, 2020; Eckerlein et al., 2019). This construct has also been negatively associated with procrastination (Bäulke et al., 2021; Kim et al., 2018, 2020) and intention to drop out (Bäulke et al., 2018; Kryshko et al., 2020). A recent longitudinal study reported a reciprocal regression relationship between global motivational self-regulation and academic adjustment (von der Mülbe et al., 2026).

Some studies have explored the possible role specific MRSs play in academic adjustment. Namely, self-efficacy enhancement seems to increase the use of (meta)cognitive strategies (Suárez-Riveiro & Fernández-Suárez, 2012) and reduce the possibility of avoiding challenge (Wang et al., 2017). In a similar fashion, self-enhancing self-talk has been shown to be significantly associated with engagement (Zenger et al., 2025). Enhancement of situational interest, cost appraisal, and self-consequating have been reported to positively predict the use of (meta)cognitive strategies and effort (Schwinger & Stiensmeier-Pelster, 2012; Wolters, 1999). On the other hand, various strategies (self-efficacy enhancement, enhancement of personal significance, cost appraisal, and self-enhancing self-talk) have been positively associated with academic achievement in elementary, middle school and high school students (Martínez-López et al., 2026; Park, 2022; Rodríguez-Guardado & Gaeta, 2020; Villar et al., 2025). The discrepancies between the use of MRSs among regular students and those with high abilities or with learning disabilities might contribute to their differing sense of belonging within the educational system. To our knowledge, no studies have explored the relationship between MRSs and self-perceived academic adjustment, nor the possible differences in that relationship among students with varying learning potential.

## 3. The Present Study

Training in learning strategies is considered a compelling and promising educational approach for supporting students both with high abilities and learning disabilities (Perry et al., 2020). Gaining a better understanding of the strengths and weaknesses of both types of students in terms of learning skills would be very valuable for the purposes of adaptive teaching. In light of the gaps identified in the literature concerning differences in the use of motivational regulation strategies across students with varying levels of learning potential, the present study aimed to address three research questions (RQs);

RQ1: Do regular students, high-ability students, and students with learning disabilities differ in their use of MRSs?
RQ2: Do the associations between motivational self-regulation strategies and academic adjustment differ across regular students, students with learning disabilities, and high-ability students?
RQ3: Are there indirect associations between students’ status (regular, high-ability, learning-disability) and academic adjustment through MRSs?

We expected that the students’ status would be associated with the use of MRSs, which, in turn, would be related to academic adjustment.

## 4. Method

### 4.1. Participants

The data were collected as part of a larger research project on self-regulated learning and academic development in adolescence. The sample consisted of 2191 students enrolled in compulsory secondary education, with a mean age of 13.86 years (*SD* = 1.21). The sample included 51.8% female students (*N* = 1136) and was distributed across four academic courses (1st grade = 19.3%, *N* = 423; 2nd grade = 27.5%, *N* = 604; 3rd grade = 26.3%, *N* = 577; and 4th grade = 26.9%, *N* = 587). Regarding the educational profile, 81.8% (*N* = 1792) were regular students (RS), 15.8% (*N* = 347) were students with learning disabilities (SWLD), and 2.4% (*N* = 52) were mathematically gifted students (MGS).

RS and SWLD were recruited from multiple secondary schools located in different towns in Spain, representing a heterogeneous range of educational and socio-geographical contexts. Students assigned to the SWLD group were identified through a sociodemographic item asking whether they received any educational support within their school. The item included examples of possible sources of support, such as the subject teacher, another teacher, or the school counseling department. Students in the MGS group were also enrolled in regular secondary schools and were identified through their participation in an extracurricular enrichment program on mathematics designed to foster mathematical talent among adolescents. Admission to the program involved a selection procedure including a mathematical problem-solving aptitude test and, for preselected candidates, an interview with the student and their family to assess their interest and commitment. All MGSs also responded to the item assessing receipt of school-based educational support. No students in the MGS group reported receiving the specific educational needs support that characterized the SWLD group. Thus, the MGS and SWLD classifications did not overlap in the present sample.

The distribution of sex and academic course across educational profiles is presented in Table 1.

Chi-square analyses indicated that group membership was not independent of sex (*χ*^2^ = 10.985, *p* = .004) or academic course (*χ*^2^ = 42.167, *p* < .001); therefore, both variables were included as covariates in subsequent analyses.

### 4.2. Measures

#### 4.2.1. Motivational Regulation Strategies

Students’ use of MRSs was measured through ten subscales derived from the Spanish adaptation of the *Motivational Regulation Survey* (Rojas-Ospina & Valencia-Serrano, 2019; Wolters & Benzon, 2013) and the *Escalas de Estrategias Motivacionales del Aprendizaje-Versión Secundaria* [Learning Motivational Strategies Scale—Secondary Version] (Suárez-Riveiro & Fernández-Suárez, 2011). Participants responded to each item using a six-point Likert scale ranging from 1 = strongly disagree to 6 = strongly agree. Sample items are listed in Table 2. Prior research conducted with Spanish student samples has provided evidence supporting the reliability and validity of both instruments (Navea-Martín & Suárez-Riveiro, 2017; Rojas-Ospina & Valencia-Serrano, 2019; Suárez-Riveiro & Fernández-Suárez, 2011). In the present study, internal consistency indices were rated as acceptable to excellent across subscales, with Cronbach’s alpha coefficients ranging from 0.72 to 0.90 and McDonald’s omega coefficients ranging from 0.73 to 0.90. Evidence of structural validity was examined through confirmatory factor analysis in Mplus 8.10, using a robust maximum likelihood estimation method. After including a small number of theoretically justified correlated residuals between conceptually overlapping items within the same subscales to account for localized item dependency, the hypothesized 10-factor model showed good fit to the data, *χ*^2^ (835) = 2448.43, *p* < .001, CFI = 0.960, TLI = 0.955, RMSEA = 0.030, 90% CI [0.029, 0.031], and SRMR = 0.038. All standardized factor loadings were statistically significant and ranged from 0.54 to 0.87.

#### 4.2.2. Academic Adjustment

Academic adjustment was assessed using a brief, four-item measure that was primarily based on the Academic Adjustment subscale of the Spanish version of the Student Adaptation to University Questionnaire (SACQ; Baker & Siryk, 1989; Rodríguez et al., 2012). Given that the original SACQ was developed for university students, the items were adapted to the secondary education context by modifying references to the university setting while preserving their original meaning. The items were as follows: “I am satisfied with the pace at which I am working”, “I am satisfied with the quality of the subjects I have”, “I like the work I do at school”, and “I get good grades”. Responses were rated on a 9-point Likert scale (where 1 = strongly disagree and 9 = strongly agree). This response format was retained from the original measure. In the present study, internal consistency was good, with Cronbach’s alpha and McDonald’s omega coefficients both equal to 0.83. A confirmatory factor analysis supported the unidimensional structure of the scale. A residual covariance between two items was freely estimated to account for item-specific shared variance, resulting in excellent model fit indices: χ^2^ (1) = 0.88, *p* = .348, TLI = 1.00, CFI = 1.00, SRMR = 0.002, and RMSEA = 0.000, 90% CI [0.000, 0.056]. All standardized factor loadings were statistically significant and ranged from 0.63 to 0.87.

#### 4.2.3. Sociodemographic Variables

Sociodemographic information was obtained through an ad hoc questionnaire assessing sex (0 = female, 1 = male), age, and course.

### 4.3. Procedure

The data were collected by two members of the research team during the 2022/2023 academic year, following approval from the university ethics committee. Written informed consent was obtained from students and their parents or legal guardians prior to participation. Students were informed that participation was voluntary and that they could withdraw at any time without consequences.

Early in the second trimester, students completed a single paper-and-pencil questionnaire composed of several sections during regular school hours. The sections relevant to the present study appeared in the following order: sociodemographic information, motivational regulation strategies, and academic adjustment; the questionnaire also included other measures that are not analyzed here. The administration of the questionnaire lasted approximately 45 min. No compensation was offered in exchange for participation.

### 4.4. Data Analysis

Preliminary analyses were carried out to calculate descriptive statistics, including means and standard deviations, as well as indicators of internal consistency reliability, namely Cronbach’s alpha and McDonald’s omega. Differences in academic adjustment among the educational profile groups were also examined using the Kruskal-Wallis test. When the omnibus test was significant, Dunn’s post hoc comparisons with Bonferroni correction were performed. These analyses were conducted using IBM SPSS Statistics for Windows, Version 29.0 (IBM Corp., Armonk, NY, USA).

The rate of missing data was below 2% overall. Although omissions were slightly more frequent among male students, cases with and without missing data did not differ significantly with regard to the study variables (all *p*s > .05), with negligible effect sizes. Therefore, missing data in the mediation models were handled using full information maximum likelihood. All analyses were performed using Mplus Version 8.10 (Muthén & Muthén, Los Angeles, CA, USA).

**Figure 1:**
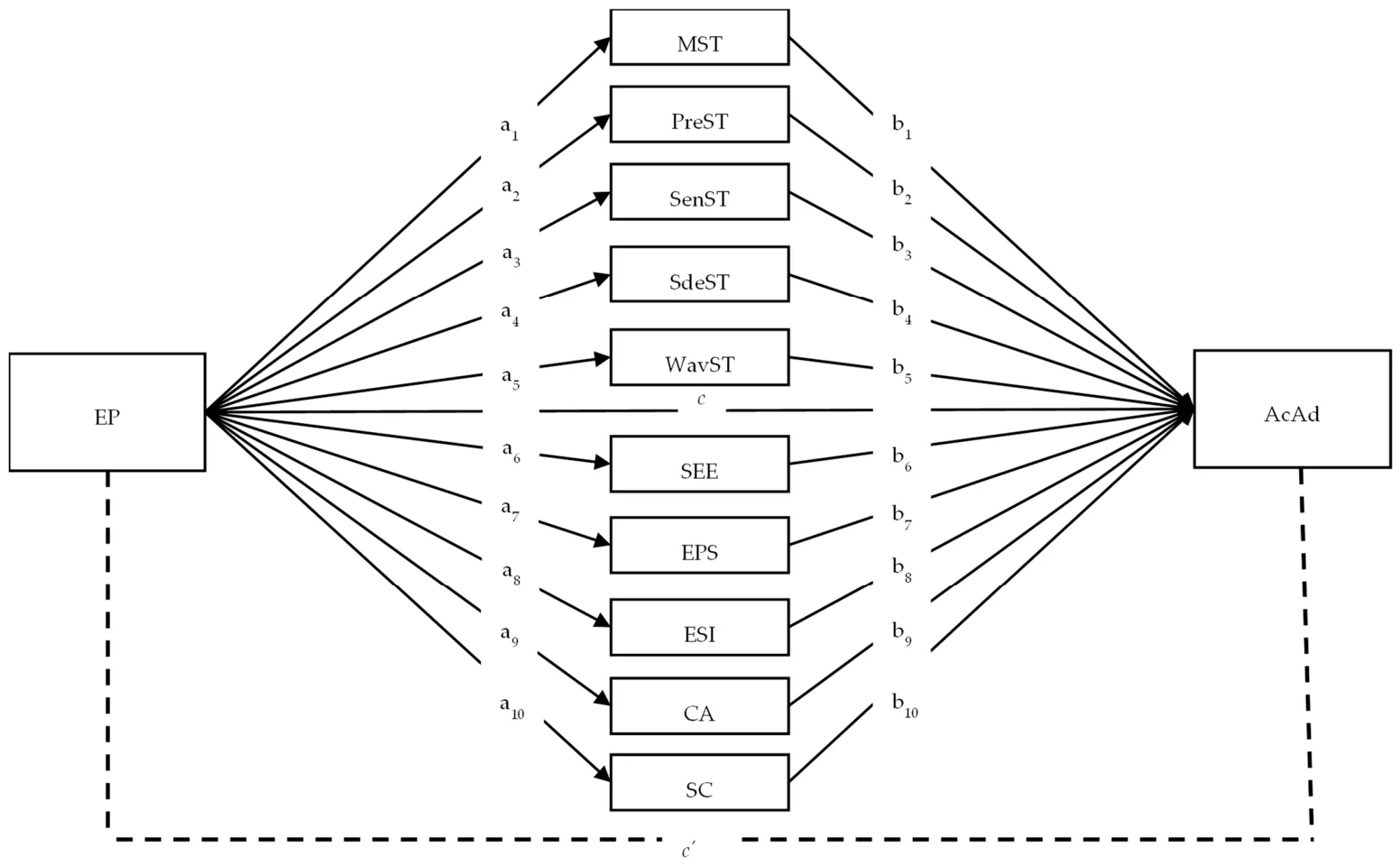
Initial path model of direct and indirect associations for each educational profile (EP). *Note.* MST = mastery self-talk; PreST = personally referred self-talk; SenST = self-enhancing self-talk; SdeST = self-defeating self-talk; WavST = work-avoidance self-talk; SEE = self-efficacy enhancement; ESI = enhancement of situational interest; EPS = enhancement of personal significance; CA = cost appraisal; SC = self-consequating; AcAd = academic adjustment.

To examine the proposed pattern of relations among educational profile, motivational self-regulation strategies, and academic adjustment, and due to the small size of the MGS group, a restricted multigroup analysis was initially performed, but the model obtained showed poor fit to the data (chi-square (86) = 724.065; *p* = .0000; RMSEA = 0.246; CFI = 0.271; TLI = 0.000; SRMR = 0.219). For this reason, three separate multiple mediation path models were then estimated. In each model, educational profile was represented by a binary indicator corresponding to one specific educational profile (1 = belonging to that profile; 0 = not belonging to that profile). Separate models were estimated for MGS, SWLD, and RS. Motivational self-regulation strategies were specified as simultaneous mediators, and sex and school grade were included as covariates of all mediators and the outcome variable. All 10 motivational regulation subscales were initially considered as candidate mediators. For each educational profile, a saturated model including all measured strategies was estimated, and a restricted model including only statistically significant mediators and structural paths was retained to obtain a more parsimonious and interpretable representation of the associations under study. The initial path model specified for each educational profile is presented in Figure 1.

School- and classroom-level identifiers were available for the RS and SWLD groups but not for the MGS group. Consequently, school- or classroom-level clustering could not be modeled consistently across the three educational profiles.

Standardized path coefficients and *R*^2^ values were estimated using robust maximum likelihood, and 95% confidence intervals were estimated for indirect effects using maximum likelihood with 10,000 bootstrap resamples. Coefficients were considered statistically significant when the corresponding confidence interval did not include zero (Preacher et al., 2007). Model fit was evaluated using the chi-square statistic, the comparative fit index (CFI), the Tucker–Lewis index (TLI), the root mean square error of approximation (RMSEA), and the standardized root mean square residual (SRMR).

## 5. Results

### 5.1. Descriptive Statistics and Internal Consistency of the Measures

Table 3 presents means and standard deviations for the study variables in the total sample and across educational profiles, together with internal consistency coefficients for the total sample.

### 5.2. Direct and Indirect Associations Across Educational Profiles

Preliminary group differences across educational profiles with respect to their academic adjustment were examined using a Kruskal–Wallis test. The group sizes for the full sample were as follows: RS = 1792; SWLD = 347; and MGS = 52; the Kruskal–Wallis test was conducted for the 2154 participants with valid academic adjustment scores. The results revealed significant differences among groups, *H* (2) = 33,95, *p* < .001, ε^2^ = 0.016 [CI = 0.007–0.260]. Dunn’s post hoc comparisons with Bonferroni correction showed significant differences between the three groups (*p* < .001), with MGS having significantly higher academic adjustment than the other two groups, and RS having significantly higher academic adjustment than SWLD.

**Figure 2:**
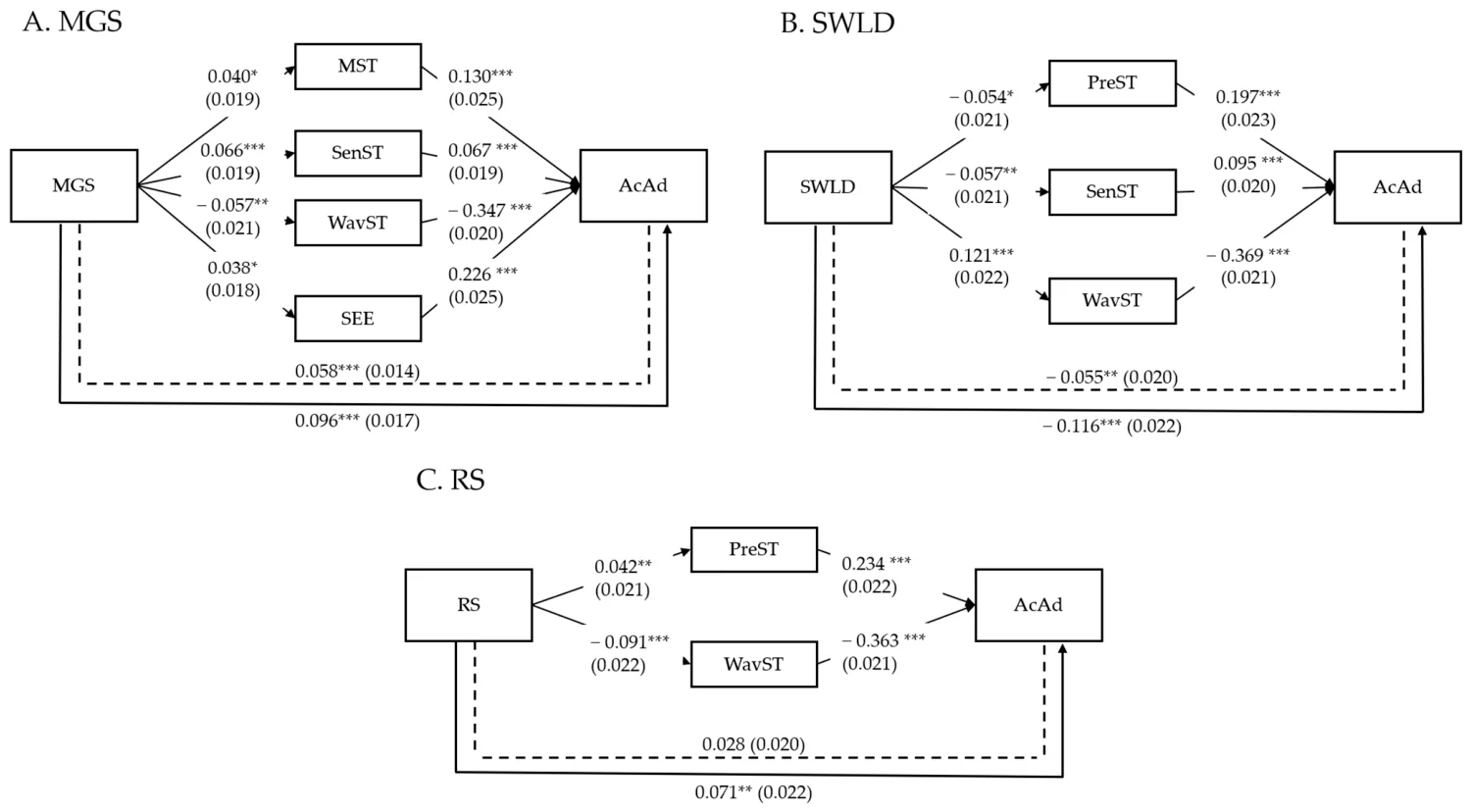
Final path models examining direct and indirect associations for (**A**) mathematically gifted students (MGS), (**B**) students with learning disabilities (SWLD), and (**C**) regular students (RS). *Note*. Standardized coefficients are shown. Standard errors are reported in parentheses. Solid lines represent total associations (c), and dashed lines represent direct associations, controlling for the strategies included in the models (c′). MST = mastery self-talk; PreST = personally referred self-talk; SenST = self-enhancing self-talk; WavST = work-avoidance self-talk; SEE = self-efficacy enhancement; AcAd = academic adjustment. * *p* < .05, ** *p* < .01, *** *p* < .001.

The three final path models examining direct and indirect associations for each group are presented in Figure 2, including all significant motivational regulation strategies. All models showed excellent fit to the data. More specifically, the model for mathematically gifted students (MGS) yielded *χ*^2^ (3) = 5.05, *p* = .168, RMSEA = 0.018, CFI = 0.999, TLI = 0.993, and SRMR = 0.008; the model for students with learning disabilities (SWLD) yielded *χ*^2^ (3) = 4.28, *p* = .233, RMSEA = 0.014, CFI = 0.999, TLI = 0.995, and SRMR = 0.009; the model for regular students (RS) yielded *χ*^2^ (1) = 1.99, *p* = .158, RMSEA = 0.021, CFI = 0.999, TLI = 0.989, SRMR = 0.007.

As shown in Table 4, the motivational pathways related to academic adjustment varied across educational profiles. For the MGS group, academic adjustment was related to various motivational regulation strategies, including mastery self-talk, self-enhancing self-talk, self-efficacy enhancement, and work-avoidance self-talk. For the SWLD group, the relevant pathways involved strategies such as personally referred performance self-talk, self-enhancing self-talk, and work-avoidance self-talk. For the RS group, two strategies were retained (personally referred performance self-talk and work-avoidance self-talk). Overall, work-avoidance self-talk emerged as the most consistent strategy involved in the indirect associations across educational profiles. The models accounted for 33.1%, 27.2%, and 26.2% of the variance in academic adjustment in MGS, SWLD, and RS, respectively.

## 6. Discussion

This study aimed to explore the possible differences in the use of motivational self-regulation strategies among students with varying learning potential (MGS, SWLD, and RS) and to examine whether the relationship between MRSs and academic adjustment differs as a function of learning-potential status. Our results indicate that high-ability status among secondary school students is positively associated with the use of mastery self-talk and self-enhancing self-talk; that regular status is positively associated with personally referred self-talk; while learning-disability status is negatively related to personally referred self-talk. All these strategies contribute to highlighting or emphasizing the utility value; that is, the usefulness of a task for achieving future outcomes or external rewards. Depending on the extent to which these goals are internalized, the aforementioned strategies will also enable the assignment of attainment value (i.e., importance and consistency with self-image) (Miele & Scholer, 2018). From the perspective of self-determination theory (Ryan & Deci, 2000), mastery self-talk and personally referred self-talk may be considered as more autonomous (less external) forms of motivational self-regulation than self-enhancing self-talk, since the latter is based on normative comparison while the other two are self-oriented, and thus representative of internalization (Hulleman et al., 2010; Reeve et al., 2008). Work-avoidance self-talk was found to be negatively associated with high-ability and regular status and positively associated with learning-disability status. Within the framework of self-determination theory, this strategy is thought to correspond to amotivation; that is, the absence of intention to engage in academic tasks, an attitude that may reflect low perceived task value and/or low self-efficacy (Ryan & Deci, 2000). Finally, we identified a positive relationship between high-ability status and the use of self-efficacy enhancement, a strategy aimed at favoring the expectation of successfully executing a task (Miele & Scholer, 2016).

According to Wolters and Benzon (2013), individuals’ preferences for certain strategies may stem from a greater degree of declarative or procedural knowledge about those strategies, or from the perception that they are particularly effective. In the absence of more extensive data regarding the use of learning motivational strategies in students with different learning potential, we can tentatively suggest that MGS might have gained more knowledge and positive experiences regarding implementation of mastery self-talk, given their greater intellectual capacity and curiosity (Kuznetsova et al., 2024). In addition, MGS may use self-enhancing self-talk as a response to the high levels of challenge and competitive pressure often associated with secondary education (Elliot et al., 2005; Yeo et al., 2009). It is likely that MGS, who are used to outperforming their peers, simply find it easy and efficient to motivate themselves by thinking about this advantage. Meanwhile, RS appear more reliant on personally referred performance self-talk, which would allow them to focus on their own progress and avoid the frustration that comes from comparing themselves to others. SWLD would likely view personally referred performance self-talk as ineffective, based on their past experiences of failure, which would likely lead them to avoid using this strategy. This interpretation would also concur with our results about the strategy of work-avoidance self-talk, which we found to be significantly and positively associated with learning-disability status. In line with the arguments presented here, it can be expected that SWLD will find it particularly difficult to appreciate the functionality of the tasks assigned in the educational system. Given their apparent lack of knowledge or poor use of other strategies that could increase their motivation, and perhaps in the absence of a sufficiently supportive classroom environment, they would choose to get by with the least amount of effort.

Both individuals’ expectations and subjective task value are considered the most proximal psychological determinants of task and activity choice and engagement in academic activities (Eccles & Wigfield, 2020). Therefore, it is reasonable to expect that strategies designed to address both motivational dimensions will be related to people’s academic adjustment. In fact, as we noted in the Introduction, there is empirical evidence supporting this relationship, and our results point in the same direction. They indicate that students with different levels of learning potential exhibit distinct tendencies when it comes to regulating their motivation. MGS seem to favor mastery and self-enhancing self-talk and self-efficacy enhancement; all these strategies are positively associated with academic adjustment. These students seem to stand out in terms of using a greater number of motivational strategies than their peers, encompassing both the motivational components of value and expectations and combining personal and normative referents of performance. This may help them to adapt more effectively to different learning conditions and to their own fluctuations in motivation. In fact, their academic adjustment scores were positively associated with their use of MRSs, except for work-avoidance self-talk, which showed a negative relationship with academic adjustment, indicating the maladaptive nature of this strategy. A direct relationship between high-ability status and academic adjustment remained after considering the indirect association of MRSs, as might be expected in light of the intellectual strengths often displayed by these individuals.

Regular-student status was not directly associated with academic adjustment once MRSs were introduced into the model, but it was indirectly related to academic adjustment through personally referred self-talk and work-avoidance self-talk. Personally referred self-talk was positively related to adjustment; this strategy could therefore be a common coping mechanism among regular students to maintain their motivation, thereby promoting their academic adjustment by focusing on their personal progress. The negative association of work-avoidance self-talk with adjustment was to be expected, given its supposed correspondence with amotivation. The use of other MRSs is likely to vary more widely among regular students, given that greater diversity is to be expected in their learning histories.

On the other hand, the self-regulation style of SWLD seems to be characterized by a greater use of work-avoidance self-talk and reduced reliance on performance self-talk, which was associated with their poorer academic adjustment. This may be explained by the fact that, when individuals adopt a work-avoidance goal orientation, success is defined in terms of minimizing effort and involvement, which reduces the likelihood of investing cognitive resources and engaging in deep learning strategies (King & McInerney, 2014). Furthermore, by avoiding the use of performance self-talk, SWLD forego normative referents for assessing their academic progress, which are, moreover, frequently encouraged in the academic environment. A direct negative relationship between SWLD status and academic adjustment remained after considering the indirect association of MRSs, as might be expected given their lower academic skills.

This study has several limitations. First, the cross-sectional design precludes conclusions about temporal ordering or causality, and the mediation models should therefore be interpreted as describing cross-sectional direct and indirect associations rather than causal processes. Second, all key variables were assessed via self-report, including motivational regulation strategies, academic adjustment, and part of the educational profile classification, which may have introduced shared method variance and reporting bias. In addition, although the brief adapted academic adjustment measure showed adequate internal consistency and a unidimensional structure in the present sample, evidence concerning its convergent and discriminant validity was not available in this dataset. Future studies should further examine the external validity of this measure in secondary education samples. Third, the educational profiles were identified using non-equivalent procedures: MGS were identified through participation in an extracurricular mathematics enrichment program, whereas SWLD were classified based on self-reported receipt of school-based educational support. Fourth, the groups were markedly unequal in size, with the MGS group representing only a small proportion of the sample. This may have limited the stability of some estimates and prevented robust testing of measurement invariance across educational profiles. Although confirmatory factor analyses supported the structure of the instruments in the full sample, measurement equivalence across RS, SWLD, and MGS could not be established; therefore, group comparisons should be interpreted with caution. Fifth, potential clustering of students within schools or classrooms could not be addressed. School-level identifiers were available for RS and SWLD, but not for the MGS group, and classroom-level identifiers were not available. Consequently, clustering could not be modeled consistently across the three educational profiles. Possible dependence among observations within educational settings should therefore be considered when interpreting the findings. Finally, although all motivational regulation subscales were initially considered, the reported models correspond to parsimonious final solutions retaining only significant predictors and indirect paths; thus, replication in independent samples would strengthen confidence in the observed pattern of associations.

## 7. Conclusions

Our findings support the role of motivational self-regulation in the academic adjustment of adolescent students with varying learning potential. These types of students seem to manifest different tendencies in the use of MRSs with unequal adaptive value. Whereas RS and MGS were associated with motivational regulation strategies positively related to academic adjustment, SWLD showed a pattern characterized by lower use of such strategies and greater use of work-avoidance self-talk.

Intervention programs, whether they are targeted at students with learning disabilities or high-ability students who are underachieving, typically include training on self-regulated learning skills (Peña-San José et al., 2026; Perry et al., 2020). However, motivational self-regulation is often overlooked, even though a failure to employ (meta)cognitive strategies is often accompanied by negative emotions and motivational beliefs, thereby undermining students’ motivation (Al-Hoorie, 2024; Efklides, 2019). Moreover, low-achieving students tend to show lower levels of self-efficacy and task values (Fong et al., 2023; Raoof et al., 2024). Teacher training programs could benefit from including content on students’ motivational self-regulation strategies and how these may influence their adaptation to academic demands. The present findings suggest that interventions should pay particular attention to reducing the use of work-avoidance self-talk and strengthening more adaptive forms of motivational regulation. In addition to fostering a classroom environment that promotes motivational autonomy, students must be equipped with effective strategies for motivational self-regulation. This approach may be particularly valuable for students who feel less academically adjusted, as is often the case for students with learning disabilities and high-ability students. The previously established association between certain cues in the learning environment and motivational beliefs may lead individuals with learning difficulties to adopt self-protective strategies, thereby neglecting their learning goals (Boekaerts, 2007). Furthermore, promoting motivational self-regulation can help students with average and high learning potential to make the most of their learning efforts and strive for excellence (Efklides, 2019).

## Figures and Tables

**Table 1:** Distribution of sex and academic course by educational profile.

Variable	Category	RS *n*	SWLD *n*	MGS *n*	Total *n*
Sex	Female	955	163	18	1136
	Male	837	184	34	1055
Academic course	1st grade	337	85	1	423
	2nd grade	470	123	11	604
	3rd grade	482	76	19	577
	4th grade	503	63	21	587
Total		1792	347	52	2191

**Table 2:** Sample items of the subscales used to measure motivational regulation strategies.

Subscale	Items	Example Item
Mastery self-talk (MST) ^1^	5	Before starting a complicated task, I set myself the goal of improving my skills and knowledge
Performance self-talk (PST)		
Personally referred performance self-talk (PreST) ^2^	5	I tell myself I need to keep studying to do well in this course
Self-enhancing self-talk (SenST) ^1^	5	I set myself the goal of doing the tasks better than others
Self-defeating self-talk (SdeST) ^1^	4	When I participate in class, I set myself the goal of trying to avoid looking incompetent to my peers
Work-avoidance self-talk (WavST) ^1^	4	I try to avoid tasks or subjects that are difficult
Self-efficacy enhancement (SEE) ^1^	5	I try to self-motivate during academic tasks by telling myself that I am doing a good job and praising my work
Enhancement of situational interest (ESI) ^2^	5	I make studying more enjoyable by turning it into a game
Enhancement of personal significance (EPS) ^2^	3	I make an effort to relate what we’re learning to my personal interests.
Cost appraisal (CA) ^1^	3	Before starting a complicated task, I usually think that accomplishment of the task will make up for the effort I have to make
Self-consequating (SC) ^2^	5	I set a goal for how much I need to study and promise myself a reward if I achieve that goal

^1^ Subscale of the Scale of Learning Motivational Strategies—Secondary Version (Suárez-Riveiro & Fernández-Suárez, 2011). ^2^ Subscale of the Spanish version of the Motivational Regulation Survey (Rojas-Ospina & Valencia-Serrano, 2019; Wolters & Benzon, 2013). *Note*. From (Martínez-López et al., 2026) CC BY 4.0.

**Table 3:** Descriptive statistics and internal consistency coefficients for the study measures.

	*M* (*SD*)	*α*	*ω*	RS	SWLD	MGS
				*M* (*SD*)	*M* (*SD*)	*M* (*SD*)
MST	3.49 (1.14)	0.817	0.819	3.46 (1.13)	3.60 (1.20)	3.65 (0.97)
PreST	4.82 (1.06)	0.850	0.848	4.84 (1.06)	4.68 (1.07)	4.88 (0.93)
SenST	3.22 (1.41)	0.882	0.878	3.24 (1.42)	3.03 (1.36)	3.82 (1.21)
SdeST	3.83 (1.49)	0.869	0.870	3.82 (1.48)	3.88 (1.51)	3.82 (1.54)
WavST	2.90 (1.36)	0.806	0.812	2.84 (1.33)	3.23 (1.42)	2.59 (1.34)
SEE	3.65 (1.41)	0.902	0.903	3.64 (1.39)	3.61 (1.51)	3.91 (1.18)
ESI	3.43 (1.27)	0.881	0.875	3.40 (1.27)	3.58 (1.33)	3.50 (1.11)
EPS	3.81 (1.22)	0.718	0.727	3.79 (1.23)	3.85 (1.17)	4.13 (1.14)
CA	3.72 (1.34)	0.850	0.853	3.70 (1.34)	3.79 (1.35)	3.69 (1.20)
SC	4.20 (1.26)	0.844	0.839	4.20 (1.25)	4.23 (1.27)	3.76 (1.42)
AcAd	5.64 (1.80)	0.830	0.832	5.68 (1.78)	5.24 (1.87)	6.60 (1.37)

*Note.* RS = regular students; SWLD = students with learning disabilities; MGS = mathematically gifted students; MST = mastery self-talk; PreST = personally referred self-talk; SenST = self-enhancing self-talk; SdeST = self-defeating self-talk; WavST = work-avoidance self-talk; SEE = self-efficacy enhancement; ESI = enhancement of situational interest; EPS = enhancement of personal significance; CA = cost appraisal; SC = self-consequating; AcAd = academic adjustment.

**Table 4:** Standardized estimates, standard errors, and 95% confidence intervals for the direct and indirect associations with academic adjustment.

	MGS								
	EP → MRSs			MRSs → AcAd			EP → MRSs → AcAd		
	*a* (SE)	*R* ^2^	95% CI	*b* (SE)	*R* ^2^	95% CI	Path	*a* × *b* (SE)	95% CI
MST	0.040 * (0.019)	0.048 ***	[0.002, 0.076]	0.130 *** (0.025)	0.331 ***	[0.081, 0.178]	*a*_1_ × *b*_1_	0.005 * (0.003)	[0.001, 0.011]
SenST	0.066 *** (0.019)	0.004	[0.028, 0.102]	0.067 *** (0.019)		[0.029, 0.103]	*a*_2_ × *b*_2_	0.004 * (0.002)	[0.002, 0.009]
WavST	−0.057 ** (0.021)	0.051 ***	[−0.097, −0.015]	−0.347 *** (0.020)		[−0.385, −0.305]	*a*_3_ × *b*_3_	0.020 ** (0.007)	[0.005, 0.034]
SEE	0.038 * (0.018)	0.008 *	[0.003, 0.072]	0.226 *** (0.025)		[0.178, 0.274]	*a*_4_ × *b*_4_	0.009 * (0.004)	[0.001, 0.017]
		SWLD							
	EP→ MRSs			MRSs → AcAd			EP → MRSs → AcAd		
	*a* (SE)	*R* ^2^	95% CI	*b* (SE)	*R* ^2^	95% CI	Path	*a* × *b* (SE)	95% CI
PreST	−0.054 * (0.021)	0.058 ***	[−0.095, −0.012]	0.197 *** (0.023)	0.272 ***	[0.152, 0.242]	*a*_1_ × *b*_1_	−0.011 * (0.004)	[−0.019, −0.002]
SenST	−0.057 ** (0.021)	0.003	[−0.096, −0.016]	0.095 *** (0.020)		[0.055, 0.134]	*a*_2_ × *b*_2_	−0.005 * (0.002)	[−0.010, −0.001]
WavST	0.121 *** (0.022)	0.064 ***	[0.079, 0.164]	−0.369 *** (0.021)		[−0.409, −0.324]	*a*_3_ × *b*_3_	−0.045 *** (0.008)	[−0.061, −0.028]
		RS							
	EP → MRSs			MRSs → AcAd			EP → MRSs → AcAd		
	*a* (SE)	*R* ^2^	95% CI	*b* (SE)	*R* ^2^	95% CI	Path	*a* × *b* (SE)	95% CI
PreST	0.042 * (0.021)	0.054 ***	[0.001, 0.084]	0.234 ** (0.022)	0.262 ***	[0.191, 0.276]	*a*_1_ × *b*_1_	0.010 (0.005)	[0.000, 0.021]
WavST	−0.091 *** (0.022)	0.058 ***	[−0.135, −0.049]	−0.363 *** (0.021)		[−0.404, −0.321]	*a*_2_ × *b*_2_	0.033 * (0.008)	[0.018, 0.050]

*Note*. EP = educational profile; SWLD = students with learning disabilities; MGS = mathematically gifted students; RS = regular students; MRSs = motivational regulation strategies; MST = mastery self-talk; PreST = personally referred self-talk; SenST = self-enhancing self-talk; WavST = work-avoidance self-talk; SEE = self-efficacy enhancement; AcAd = academic adjustment. Standardized coefficients (β) and *R*^2^ values are reported from the MLR analyses; 95% confidence intervals are reported from the ML bootstrap analyses. In the EP → MRSs section, *R*^2^ refers to the explained variance of each motivational regulation strategy included in the model. In the MRSs → AcAd section, *R*^2^ refers to the explained variance of academic adjustment. * *p* < .05, ** *p* < .01, *** *p* < .001.

## Data Availability

The data (anonymized, with no identifying information) are available at https://osf.io/rv8b5/overview (accessed on 14 August 2026).

## Abbreviations

The following abbreviations are used in this manuscript:

AcAd	academic adjustment
CA	cost appraisal
EPS	enhancement of personal significance
ESI	enhancement of situational interest
MGS	mathematically gifted students
MRSs	motivational regulation strategies
MST	mastery self-talk
PreST	personally referred self-talk
RS	regular students
SC	self-consequating
SdeST	self-defeating self-talk
SEE	self-efficacy enhancement
SenST	self-enhancing self-talk
SRL	self-regulated learning
SWLD	students with learning disabilities
WavST	work-avoidance self-talk
